# Wnt Pathway in Pancreatic Development and Pathophysiology

**DOI:** 10.3390/cells12040565

**Published:** 2023-02-09

**Authors:** Tiziana Napolitano, Serena Silvano, Chaïma Ayachi, Magali Plaisant, Anette Sousa-Da-Veiga, Hugo Fofo, Benjamin Charles, Patrick Collombat

**Affiliations:** 1 DiogenX, 180 Avenue du Prado, 13008 Marseille, France; 2 Université Côte d’Azur, CNRS, Inserm, iBV, 06000 Nice, France

**Keywords:** Wnt pathway, pancreas, β-catenin, embryonic development, β-cells, diabetes

## Abstract

The pancreas is an abdominal gland that serves 2 vital purposes: assist food processing by secreting digestive enzymes and regulate blood glucose levels by releasing endocrine hormones. During embryonic development, this gland originates from epithelial buds located on opposite sites of the foregut endoderm. Pancreatic cell specification and maturation are coordinated by a complex interplay of extrinsic and intrinsic signaling events. In the recent years, the canonical Wnt/β-catenin pathway has emerged as an important player of pancreas organogenesis, regulating pancreatic epithelium specification, compartmentalization and expansion. Importantly, it has been suggested to regulate proliferation, survival and function of adult pancreatic cells, including insulin-secreting β-cells. This review summarizes recent work on the role of Wnt/β-catenin signaling in pancreas biology from early development to adulthood, emphasizing on its relevance for the development of new therapies for pancreatic diseases.

## 1. Introduction

The pancreas is a digestive gland able to orchestrate endocrine and exocrine physiological responses due to the anatomical and functional interplay of its three main components: the islets of Langerhans, acini and ducts. Acinar cells are specialized in the synthesis, storage and release of digestive enzymes for intestinal digestion. These enzymes are collected by the pancreatic ductal network to be finally conveyed into the duodenum [1,2]. Ductal cells not only deliver enzymes into the duodenum, but also actively secrete sodium bicarbonate that neutralizes the acidic pH of the gastric contents delivered to the intestine from the stomach. This neutralizing activity of the ductal secretion is crucial, as pancreatic enzymes display optimal activity at neutral pH [3]. Scattered throughout the pancreas parenchyma, pancreatic endocrine cells are organized into highly vascularized clusters termed islets of Langerhans. These cells are classified into β-, α-, δ-, PP-, and ε-cells, secreting insulin, glucagon, somatostatin, pancreatic polypeptide, and ghrelin, respectively [4]. The main role of the endocrine pancreas is to ensure a homeostatic control of blood glucose levels by secreting insulin (acting to decrease glycemia) and glucagon (whose role is to increase glycemia). 

Glucose metabolism is severely impaired in diabetic patients. Indeed, type I diabetes mellitus (T1DM) is a chronic autoimmune disease characterized by a severe deficiency in insulin secretion as a result of an auto-immune-mediated pancreatic β-cells loss [5]. Conversely, Type II diabetes mellitus (T2DM) is associated with the dysregulation of carbohydrate, lipid, and protein metabolism, resulting from impaired insulin secretion, insulin resistance or a combination of both [6]. Currently, T1DM treatment mainly consists in the administration of exogenous insulin, while T2DM management relies on several drugs aiming at enhancing insulin action and/or secretion, in order to overcome the insulin resistance.

Despite impressive advances in diabetes treatment, glycemic control for most diabetics is not fully achieved and their life expectancy/quality of life remain altered when compared to the general population [7]. Towards the ultimate goal of discovering new strategies to cure and prevent diabetes and despite impressive progresses, a deep understanding of molecular mechanisms regulating pancreas development remains required. Indeed, Genome-Wide Association (GWA) studies revealed that several polymorphisms associated with high risks to develop diabetes lie on genes playing a key role in pancreatic cells differentiation [8,9]. Furthermore, the identification of the factors belonging to the intricate regulatory network coordinating pancreas development could further improve stem cell differentiation strategies or pave the path for the conversion of pancreatic cells into functional β-cells [10,11,12,13,14,15]. 

The Wnt pathway represent a highly evolutionary conserved signaling pathway required in most embryonic developmental processes, both in invertebrates and vertebrates. Accordingly, this pathway also belongs to the complex system of signaling processes that orchestrate pancreas organogenesis [16,17]. Wnt proteins have indeed been shown to regulate pancreatic β-cell proliferation and glucose-stimulated insulin secretion [18,19].

**Figure 1:**
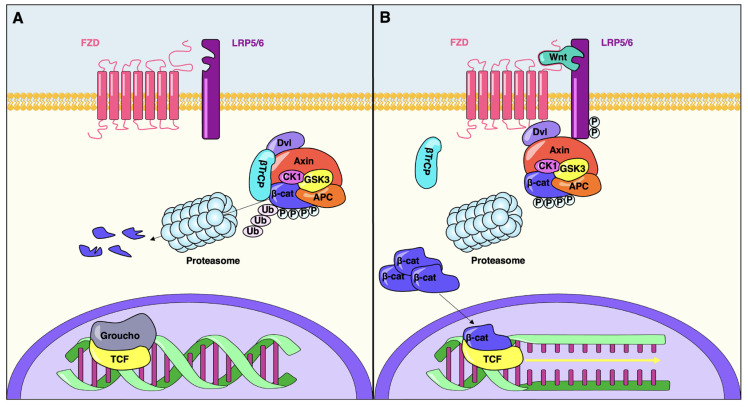
Canonical Wnt signaling pathway. (**A**) In the absence of Wnt ligands, cytoplasmic β-catenin is phosphorylated by the destruction complex, which includes Axin, adenomatosis polyposis coli (APC), glycogen synthase kinase 3 (GSK3) and casein kinase 1 (CK1). Phosphorylation of β-catenin within this complex by CK1 and GSK3 targets it for ubiquitination and subsequent proteolytic destruction by the proteosomal machinery. In the absence of nuclear β-catenin, TCFs engage with Groucho, which prevents the transcription of Wnt target genes. (**B**) The interaction of Wnt with its receptors FZD and LRP5/6 leads to the binding of Axin with phosphorylated LRP5/6 and the stabilization and nuclear accumulation of β-catenin. β-catenin is subsequently translocated into the nucleus where it interacts with TCFs and upregulate the transcription of Wnt target genes.

Extra-cellular Wnt proteins activate three intra-cellular transduction cascades: the canonical Wnt/β-catenin pathway, the non-canonical planar cell polarity pathway and the Wnt/Ca^2+^ pathway [20,21,22]. Wnts have been shown to play a pivotal role in regulating pancreatic cell development and function mainly through the canonical pathway (schematized in Figure 1), onto which this review will focus.

## 2. The Canonical Wnt Signaling Pathway

Wnt glycoproteins activate a complex signaling pathway that plays a critical role both during embryonic development and adult tissue homeostasis, by regulating cell proliferation, cell polarity and cell fate determination. The *Wnt1* gene was first discovered in two independent studies in *Drosophila* and mouse. The fly *Wingless* (*wg*) was identified as a segment polarity gene during larval development while *Int-1* was found to be a protooncogene responsible for virally induced mammary tumors in mice [23,24]. *Wg* and *Int-1* were later shown to encode homologous proteins and their names were merged into “Wnt” [25,26]. 

Genome sequencing has revealed that mammals encode 19 Wnt ligands, which are highly conserved, cysteine rich secreted glycoproteins [27]. Wnt signals are generally considered as classical morphogens, diffusible molecules that elicit long-range concentration-dependent changes in gene expression and cellular behavior. However, Wnts are post-translationally modified during their synthesis through cysteine palmitoylation [28,29,30]. Besides being essential for Wnt biological activity, this modification strongly reduces Wnt solubility, possibly restricting its diffusion through the extracellular space. Accordingly, Wnt signaling has been observed to mostly occur between neighboring cells in most tissues, the ability of Wnts to act as long-range signaling molecules remaining a matter of debate [31]. 

The main molecular event controlled by the canonical Wnt pathway is the regulation of cytosolic β-catenin stability by inhibiting the activity of the destruction complex (DC). In this complex, the scaffold protein Axin interacts with two serine-threonine kinases GSK3 and CK1, the tumor suppressor protein APC and β-catenin. In the absence of a Wnt stimulus, β-catenin interacts with the destruction complex prior to being sequentially phosphorylated by CK1 (at Ser45) and GSK3 (at Ser33/37/Thr41), ubiquitinated by the β-TrCP ubiquitin E3 ligase complex, and finally degraded by the proteasome (Figure 1) [32]. 

Upon secretion, Wnt ligands interact with a receptor complex made out of two proteins present on the cell surface (Figure 1): the Frizzled seven-pass transmembrane receptors (FZD) and the LDL receptor-related proteins 5 and 6 (LRP5/6) [33,34]. Wnt binding leads to heterodimerization of FZD and LRP5/6, followed by conformational changes that ultimately result in the phosphorylation of LRP5/6 intracellular domain and the docking of Axin [35,36,37]. In other words, the main outcome of Wnt receptors engagement is the recruitment of the DC to the cell membrane, the subsequent inhibition of its activity and the consequent accumulation of stable, non-phosphorylated β-catenin in the cytoplasm and nucleus [38,39]. 

TCF transcription factors represent the main effectors of the canonical Wnt pathway [40,41]. They repress gene expression by interacting with Groucho proteins and promoting histone deacetylation and chromatin compaction [42,43]. Following its translocation into the nucleus, β-catenin displaces Groucho and forms a complex with TCFs, transiently converting them into transcriptional activators [44]. 

Besides Wnt proteins, several secreted molecules able to positively and negatively regulate canonical Wnt pathway have been identified. Among these, R-spondin proteins have emerged as crucial agonist of the Wnt signaling cascade in different vertebrate species [45,46,47]. While the detailed molecular mechanisms underlying their activity need to be further elucidated, it has become clear that R-spondins promote Wnt signaling by synergizing with Wnt proteins to stabilize the levels of cytosolic β-catenin [48].

Additional factors may also modulate the canonical Wnt pathway. For instance, Norrin proteins are able to directly bind FZD4 and activate the canonical Wnt signaling pathway in an LRP5/6-dependent fashion [20,49]. Furthermore, Wnt activity is regulated by extracellular inhibitors. The Dickkopf (Dkk) and Sclerostin/SOST proteins represent two families of specific Wnt inhibitors that antagonize Wnt signaling by direct interacting with the LRP5/6 receptor [27,50]. Finally, sFRPs (secreted Frizzled related protein) and WIF (Wnt inhibitory factor) have been shown to antagonize both canonical and non-canonical Wnt signaling upon integration with Fzd receptors and/or Wnt ligands [27,51].

**Figure 2:**
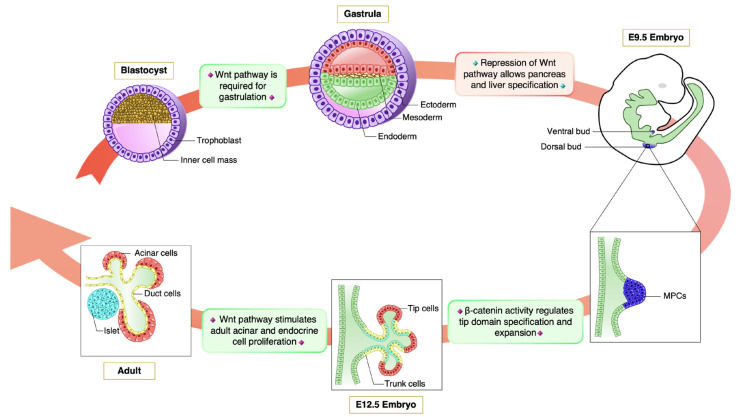
Schematic representation of canonical Wnt pathway activity during pancreas organogenesis. The blastocyst comprises an outer layer of trophoblats and an inner collection of cells termed the inner cell mass (ICM) cells. During gastrulation, the ICM is reorganized into three germ layers: the endoderm, the ectoderm and the mesoderm. At this stage, Wnt/β-catenin signaling activity plays a critical role for the progression of gastrulation and formation of mesoderm and endoderm [16,52,53,54,55,56,57,58,59,60]. Following gastrulation, the endoderm germ layer forms a primitive gut tube from which pancreatic buds emerge. While pancreas specification relies on the repression of the Wnt/β-catenin pathway [61,62,63], subsequent expansion and compartmentalization of multipotent progenitor cells strongly depends on the pathway being active [64,65,66,67]. Finally, Wnt/β-catenin signaling regulates homeostasis and expansion of terminally differentiated adult acinar and endocrine cells [68,69].

Canonical Wnt pathway modulates pleiotropic biological functions during organ development. In the recent years, this complex signaling cascade has come under close scrutiny for its role in pancreas organogenesis. The results of these investigations are summarized in the next paragraph and in Figure 2.

## 3. Wnt Pathway in Pancreas Organogenesis

In human and mouse blastocyst, cells of the inner cell mass (ICM) differentiate into a two-layered tissue containing the epiblast (dorsal) and the hypoblast (ventral), which are together known as the bilaminar disc. During gastrulation, epiblast cells are subsequently allocated to the three definitive germ layers: the ectoderm, the mesoderm and the endoderm. The onset of gastrulation is marked by the formation of the primitive streak, a linear band of thickened epiblast that appears at the caudal end of the embryo. As gastrulation progresses, pluripotent epiblast cells undergo an epithelial-to-mesenchymal transition to migrate into the primitive streak, emerging as either mesoderm or endoderm, while the remaining epiblast cells will transform into ectoderm [70,71].

Genetic studies in mouse demonstrated that canonical Wnt pathway belongs to a signaling network that orchestrate the generation of the three germ layers. Decades of research in mouse embryo have revealed that gastrulation is sustained by a positive feedback loop involving Wnt3, bone morphogenetic protein (BMP) and Nodal signaling [16,52]. In mice lacking *Wnt3a* ligand, as wells as in *Lrp5*/*Lrp6* double knockouts, *Lef1*/*Tcf1* double mutants and *Ctnnb1* (gene encoding the protein β-catenin) null animals, primitive streak formation is severely impaired, indicating that the Wnt pathway is required for mesoderm and endoderm formation [53,54,55,56,57,58,59]. In a more recent work, Tortelote at al. inactivated *Wnt3a* specifically in the epiblast and demonstrated that this gene is not essential for the initial stages of gastrulation and the establishment of the primitive streak, but rather for the maintenance and finalization of gastrulation processes [60]. 

Once the primary germ layers are established, the endoderm undergoes a complex series of morphogenic modifications to give rise to the primitive gut tube, divided into foregut, midgut and hindgut. In the mouse, the first indication of pancreas development appears around embryonic day 9 (E9), with the emergence of epithelial buds on opposing sides of the foregut endoderm. These buds are composed of multipotent progenitor cells (MPCs), labeled by the expression of *Pdx1*, *Ptf1a* and *Sox9* genes. Subsequently, the developing pancreatic epithelium grows and forms multiple branched protrusions. Cells in the tip domain give rise to acinar cells, while inner cells composing the trunk domain differentiate into either endocrine or ductal cells [72,73].

In Xenopus gastrula embryos, repression of canonical Wnt pathway is required to maintain foregut identity and allows both pancreas and liver specification. Indeed, forcing β-catenin activity in the anterior endoderm has been shown to indirectly downregulate *hhex*, one of the earliest foregut markers, leading to ectopic intestinal gene expression in the foregut domain [61]. Accordingly, β-catenin activation in mouse MPCs has been reported to severely impair pancreas formation, reduce epithelial branching and markedly compromise the differentiation of endocrine and exocrine lineages [62]. Besides, constitutively stabilized β-catenin in the Pdx1 domains was found to redirect the developmental fate of MPCs towards a gastrointestinal lineage [62]. These results are consistent with other studies demonstrating that the overexpression of *Wnt1* and *Wnt5a* in the developing pancreas epithelium greatly compromise pancreas development, strongly reducing the size of the organ or causing complete pancreas agenesis [63]. 

Recently, β-catenin has been suggested to play a role in MPCs compartmentalization in developing mouse pancreas. Specifically, upon deletion of *Ctnnb1* in MPCs, the pancreatic epithelium at E12.5 has been found to acquire a trunk phenotype devoid of branching tips [64]. Consequently, pancreas specimens from *Ctnnb1* conditional knockout animals were markedly hypoplastic and histologically characterized by a dramatic paucity of acinar cell mass. Several independent studies revealed that the canonical Wnt pathway also plays a key role in stimulating the proliferation of pancreatic progenitors throughout the developing organ [65,66,67]. Therefore, β-catenin appears to be required for the differentiation and the expansion of pancreatic acinar compartment.

By E15.5, most tip cells have undergone acinar cell differentiation and the subsequent expansion of the acinar domain is largely driven by cell proliferation until weaning [74]. Adult acinar cells, however, show a low basal proliferative rate and appear to exhibit poor regenerative capacity [74,75]. Using an inducible conditional knockout mouse model to specifically ablate *Ctnnb1* in acinar cells, Keefe et al. demonstrated that β-catenin function is also crucial for post-natal acinar cell proliferation. Specifically, the lack of *Ctnnb1* was shown to compromise the expansion of the acinar cell mass in infants, juveniles, and adult mice in physiological conditions or upon caerulein-induced pancreatitis [68]. Altogether, these studies highlight a continuous requirement for β-catenin in the specification and maintenance of acinar cell mass, extending from embryonic organogenesis through expansion and homeostasis in the juvenile and adult organ, as well as regeneration following injury. 

While tip cells differentiate into acinar cells, the trunk domain forms a network of tubules, referred to as the primitive ducts. Within this epithelium, a subset of cells activates the expression of *Neurogenin3*, delaminate from the primitive duct and differentiate in mature pancreatic endocrine cells [72,73]. Of note, the competence of endocrine precursors to produce different endocrine cell types is temporally controlled, as they first differentiate into α-cells, then β-cells followed by δ- and PP-cells [72,73]. 

During pancreas morphogenesis in zebrafish and mouse, both Wnt5 ligand and Fzd-2 receptor have been suggested to be required for insulin-expressing cell migration from the duct [76]. Interestingly, the deletion of *Ctnnb1* in MPCs was associated with a decreased number of islets of Langerhans in newborn mice, these results collectively suggesting that canonical Wnt pathway may be involved in the expansion and/or survival of endocrine cells during development [77]. However, several studies failed to detect any change in islet area or function in the absence of *Ctnnb1* [65,66]. These discrepancies may result from different quantitative approaches or different Cre mouse lines. Accordingly, several Pdx1-Cre mouse strains exhibit significant differences in terms of spatial and temporal activity of the Cre recombinase as well as its recombination efficiency [78]. For instance, opposite results have been reported in studies using in parallel two Pdx1-Cre lines to activate β-catenin, demonstrating that the role of this signaling protein in pancreas specification varies depending on the developmental stages. Particularly, stabilizing β-catenin activity early in development abolishes organ formation. By contrast, inducing the stabilized form of β-catenin at a later time point in pancreas development enhanced proliferation and caused a dramatic increase in pancreas size [79]. 

Recently, it has been demonstrated that, in the absence of *Ctnnb1*, MPCs fail to form a tip domain, acquire trunk cell features and prematurely generate early endocrine precursor cells fated to become α-cells. The resulting impoverished pool of endocrine precursors were unable to generate the full complement of β-cells, causing reduction in β-cell mass at late developmental stages. The authors therefore proposed that β-catenin plays a key role in the specification of both acinar and endocrine cell lines [64]. 

Altogether, these studies demonstrate that the precise spatial and temporal activation of Wnt target genes plays a pivotal role in pancreas development. Figure 2 highlights the stages of mouse pancreas organogenesis regulated by canonical Wnt pathway: in the early embryogenesis, Wnt/β-catenin signaling activity is critical for the formation of the primitive streak and progression of gastrulation. Subsequently, the inhibition of the pathway is required for proper pancreatic specification during early endoderm development. Interestingly, the growth and differentiation of fetal pancreatic cells, as well as the proliferation of adult cells, rely on β-catenin function. 

Fully understanding the molecular mechanisms underlying endoderm specification raises increasing academic and practical interest. Indeed, temporally appropriate manipulation of these pathways will be required to further improve the protocols aiming at differentiating human stem cells into endoderm and its derivatives, such as mature β-cells. 

## 4. Relevance of Wnt Signaling Cascade in β-Cell Biology

Pancreatic β-cells represent the core of glucose homeostasis, as they secrete the only hormone able to directly decrease blood glucose levels. Therefore, loss or disfunction of this cell subtype is sufficient to cause diabetes mellitus. For this reason, dissecting the molecular mechanisms underlying adult β-cell physiology, replication and secretion has received considerable attention by research groups aspiring to develop novel therapies for diabetes. 

Besides its role during pancreas morphogenesis, β-catenin has been widely reported to modulate adult β-cell proliferation, survival and function. Post-natal expressions of 5 Wnt ligands and 8 members of FZD receptors have been initially observed in mouse whole pancreas samples [63]. Thorough transcriptomic analyses recently revealed that 15 Wnt subtypes, together with FZD, LRP5 and LRP6 receptors are expressed specifically in mouse adult endocrine cells [80]. Seemingly, a RT-PCR screening detected at least 7 Wnt genes and 3 FZD genes in purified adult human islets obtained from cadaveric nondiabetic donors [81]. 

Stimulating β-cell proliferation might represent a straightforward and effective way to counteract the β-cell loss occurring in diabetic patients [82]. However, the adult endocrine pancreas has been shown to display a slow cell turnover [83]. Nevertheless, several reports suggest that differentiated β-cells might be responsive to pharmacologically active molecules promoting their expansion or inhibiting cell death [84]. Glucagon-like peptide-1 (GLP-1) is a gut incretin hormone that enhances glucose disposal by controlling glucose-stimulated insulin release. Besides their insulin secretagogue action, both GLP-1 and its long-acting agonist exendin-4 have been observed to promote β-cell proliferation [85,86]. Importantly, Liu and colleagues observed that exendin-4 activates the Wnt pathway both in the INS-1 β-cell line and isolated mouse islets of Langerhans. In an effort to investigate the mitotic activity that GLP-1 exerts on β-cells, the authors established that the interaction between exendin-4 and GLP-1 receptor (GLP-1R) leads to β-catenin stabilization through PKA-mediated phosphorylation in INS-1 cells [87]. This GLP-1/GLP-1R/PKA axis finally leads to a TCF7L2-dependent up-regulation of several proliferative genes, including *c-myc* and *cyclin D1* [87]. 

Following early neonatal period, murine β-cell proliferation rate dramatically declines resting at approximately 1% throughout adulthood [88]. Thus, these cells appear to lack the regenerative capacity required to compensate the severe β-cell loss occurring in diabetes pathogenesis. Intriguingly, purified Wnt3a has been demonstrated to increase β-cell replication not only in an immortalized β-cell line (MIN6 cells), but also in murine isolated islets [69]. These results indicate that targeting Wnt signaling pathway might be able to reawaken the regenerative potential of adult pancreatic β-cells. 

In addition to its ability to sustain β-cell proliferation in vitro, several lines of evidence indicate that Wnt signaling cascade might activate β-cell regeneration also in living animals. Particularly, expression of a stabilized form of β-catenin specifically in β-cells led to an increase in proliferation marker expression in adult murine β-cells and a consequent increase in β-cell mass, in vivo. Accordingly, constitutive activation of β-catenin was associated with lower basal glycemia, improved glucose handling and higher blood insulin levels, reflecting increased β-cell volume [69]. Equally interesting was the stimulation of β-cell regeneration in neonatal rats upon activation of Wnt/β-catenin pathway. In this model, *TCF7L2* downregulation reduced β-cell mass and altered β-cell regeneration upon streptozotocin-induced diabetes. Conversely, activation of Wnt signaling by inhibition of GSK-3β led to a dramatic improvement of β-cell mass in diabetic neonatal rats [89].

Proliferation of mature, terminally differentiated β-cells at high rate has been observed only under few physiological circumstances. For instance, in high-fat feeding obesity models, the increased demand for insulin is accompanied by an adaptive expansion of the β-cell population. An in-depth analysis of the molecular link between obesity, insulin resistance and β-cell hyperplasia may offer a viable option for the restoration of the β-cell mass after diabetic loss. Interestingly, an increase in β-cell mass together with an augmentation of intrapancreatic content of TCF7L2 was demonstrated in mice following an 8-week high-fat feeding course [90]. Conversely, *TCF7L2* expression levels decreased after 12 weeks of high-fat feeding, when hyperglycemia and β-cell failure occur. Additional studies demonstrated that hyperplastic islet cells in animals fed with high-fat diet for 60 days showed β-catenin translocation into β-cell nuclei, together with increased expressions of *β-catenin*, *cyclin D1*, *cyclin D2* and *c-myc* both at the gene and protein levels [91]. These studies indicate that the canonical Wnt pathway might play a role in the development of the compensatory β-cell hyperplasia observed in obese mice.

T2DM results from impaired insulin sensitivity and β-cell function. Hence, identifying novel insulin secretagogues might represent a valuable tool to prevent or delay this form of diabetes. Notably, both Wnt3a- and Wnt5a-conditioned media have been found to markedly stimulate glucose-induced insulin secretion from isolated murine islets [92]. This activity was abolished by the addition of a FZD antagonist or genetic ablation of *LRP5*. Also, and more importantly, loss of LRP5 caused impaired glucose tolerance and insulin secretion without reducing β-cell number, in vivo. Altogether, these data show that Wnt pathway not only promotes β-cell mitosis but is also crucial to maintain physiological β-cell function.

Human adult β-cells proliferate at a lower rate compared to rodent cells and are poorly responsive or unresponsive to the numerous mitogens, growth factors, and nutrients able to induce expansion in rodent models [93]. Therefore, finding strategies to expand human β-cells represents a major challenge in diabetes research. In this contest, Aly et al. described an approach to engage Wnt signaling by incubating human islets with a condition medium from L-cells that constitutively express Wnt3a, R-spondin-3 and Noggin supplemented with RhoA/Rock inhibitors. Impressively, this medium significantly increased the number of Ki67-extressing β-cells by 20-fold, while preserving the β-cell phenotype and insulin secretion [94].

**Figure 3:**
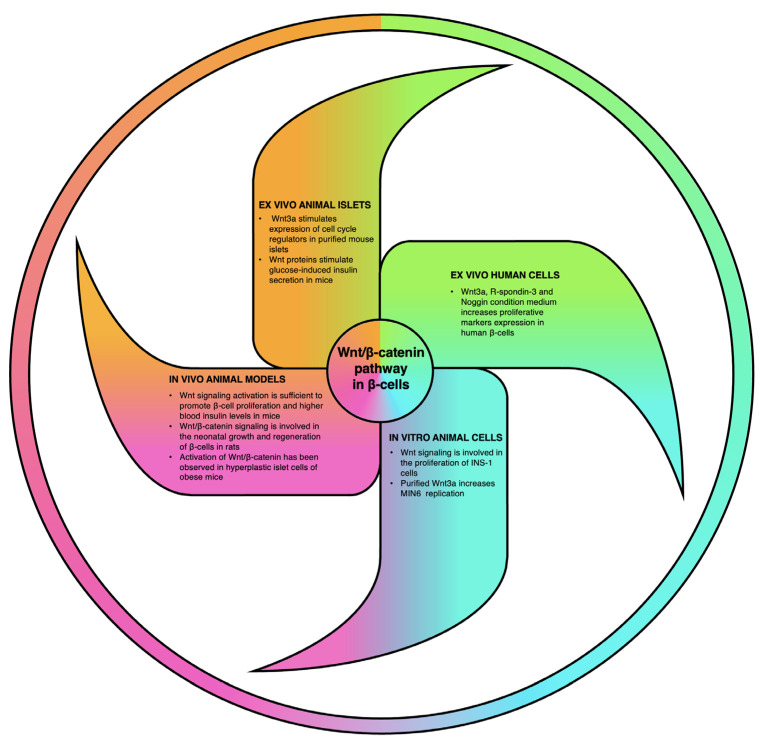
The “β-cell Wntmill”: Wnt/β-catenin pathway has been shown to play a role in β-cell proliferation and function in several experimental settings. The activation of this pathway has been demonstrated to induce β-cell proliferation and insulin secretion in immortalized cells in vitro [69,87], in isolated mouse islets ex vivo [69,92] and in vivo [69]. Also, activation of Wnt signaling is involved in β-cell regeneration and expansion in in diabetic neonatal rats [89] and in high-fat-induced diabetic mice [90,91]. Finally, Wnt ligands appear also to increase proliferation of human β-cells ex vivo [94].

Figure 3 summarizes all the experimental approaches used to understand the role of Wnt/ β-catenin signaling pathway in pancreatic β-cell proliferation and function and the main results obtained. In light of these works, it appears evident that Wnt pathway impacts pancreatic β-cell viability and hormone secretion. A large body of data indicates that activation of this pathway can stimulate β-cell proliferation in immortalized cell lines, isolated endocrine cells and animal models. As manipulation of Wnt signaling seems to play a role also in human β-cell expansion, a further understanding of the role of β-catenin in pancreatic endocrine cells might unravel novel targets in the design of therapeutic strategy to counter diabetes.

## 5. Concluding Remarks

Wnt/β-catenin pathway plays instrumental roles in the development of virtually all tissues and organ systems. In this review, we summarized the main discoveries revealing the key role that this signaling cascade plays in endoderm formation, pancreas organogenesis and pancreatic cell specification. These data might find important applications in the design of experimental protocols for β-cell regeneration. For instance, several studies showed that canonical Wnt signaling cascade needs to be inhibited during early stages of pancreas specification, indicating that blocking this pathway may be required to efficiently derive β-cells from stem cells, in vitro.

The loss of functional β-cells is a crucial event in the development of chronic hyperglycemia in both T1DM and T2DM. Hence, pharmacological activation of β-cell expansion might represent a suitable strategy to overcome β-cell deficiency in a diabetic context. Therefore, intensive search has been dedicated to identifying signaling molecules that could be manipulated to stimulate β-cell growth, in vivo. It is becoming progressively evident that the Wnt/β-catenin pathway impacts pancreatic endocrine cell proliferation, secretion and viability. Further confirmations supporting the role of β-catenin pathway in islet functions derive from human genetic studies showing that a polymorphism of TCF7L2 is associated with an increased risk of developing T2DM in numerous populations [95,96,97,98,99]. 

As β-catenin display both β-cell regenerative and insulin secretagogue activities, developing Wnt-stimulating therapeutic strategies to treat diabetic patients could become extremely tempting. However, the Wnt pathway is involved in maintaining adult tissue homeostasis in several systems. Therefore, the clinical use of reagents that modulate β-catenin activity requires careful safety assessment. Nevertheless, drugs that have already been approved for diabetes management might exert their therapeutic effect also through modulation of Wnt pathway. As previously mentioned exendin-4 seems to act not only as a GLP-1R agonist, but it appears to also induce β-cell proliferation by stabilizing β-catenin, consequently activating several Wnt target genes. Hence, pharmacological activation of canonical Wnt pathway might represent a reliable option to develop more effective treatments for β-cell failure for the clinical management of both T1DM and T2DM.

Due to their hydrophobicity, Wnt proteins might not be ideal drug candidates. However, several Wnt agonists have become available and are already bearing hopes for the development of novel regenerative therapies [100]. For instance, administration of the Wnt agonist (2-amino-4-[3,4-(methylenedioxy)benzylamino]-6-(3-methoxyphenyl)pyrimidine) has been shown to increase hepatocyte proliferation, decrease inflammation, apoptosis and necrosis in a hepatic ischemia rat model [101]. Also, a synthetic Wnt/β-catenin signaling activator has been found to induce hair regrowth in mice and increase hair length in human hair follicles [102]. Importantly, a monoclonal antibody (Romosozumab) that inhibits the Wnt signaling antagonist sclerostin has recently been approved for osteoporosis treatment, as two trials demonstrated that it reduced the rate of vertebral fracture [103]. The identification of a natural or recombinant molecule capable to promote pancreatic β-cell regeneration might represent one of the next major breakthroughs in the field of diabetes research.
